# Development of lower limb range of motion from early childhood to adolescence in cerebral palsy: a population-based study

**DOI:** 10.1186/1741-7015-7-65

**Published:** 2009-10-28

**Authors:** Eva Nordmark, Gunnar Hägglund, Henrik Lauge-Pedersen, Philippe Wagner, Lena Westbom

**Affiliations:** 1 Department of Health Sciences, Division of Physiotherapy, Lund University, SE-221 00 Lund, Sweden; 2 Hospital for Children and Adolescents, Lund University Hospital, SE-221 85 Lund Sweden; 3 Department of Orthopaedics, Lund University Hospital SE-221 85 Lund, Sweden; 4 National Competence Centre for Musculoskeletal Disorders, Lund University Hospital, SE-221 85 Lund, Sweden; 5 Department of Clinical Sciences, Division of Paediatrics, Lund University, Lund, Sweden

## Abstract

**Background:**

The decreasing range of joint motion caused by insufficient muscle length is a common problem in children with cerebral palsy (CP), often worsening with age. In 1994 a CP register and health care programme for children with CP was initiated in southern Sweden. The aim of this study was to analyse the development of the passive range of motion (ROM) in the lower limbs during all the growth periods in relation to gross motor function and CP subtype in the total population of children with CP.

**Methods:**

In total, 359 children with CP born during 1990-1999, living in the southernmost part of Sweden in the year during which they reached their third birthday and still living in the area in the year of their seventh birthday were analysed. The programme includes a continuous standardized follow-up with goniometric measurements of ROM in the lower limbs. The assessments are made by each child's local physiotherapist twice a year until 6 years of age, then once a year. In total, 5075 assessments from the CPUP database from 1994 to 1 January 2007 were analysed.

**Results:**

The study showed a decreasing mean range of motion over the period 2-14 years of age in all joints or muscles measured. The development of ROM varied according to GMFCS level and CP subtype.

**Conclusion:**

We found a decreasing ROM in children with CP from 2-14 years of age. This information is important for both the treatment and follow-up planning of the individual child as well as for the planning of health care programmes for all children with CP.

## Background

Muscle shortening and a decreased passive range of motion (ROM) are common in children with cerebral palsy (CP) [1]. Decreased ROM may cause several problems related to body function and structure, such as hip dislocation, windswept deformity and scoliosis. It is one factor contributing to the deterioration of functional skills, such as walking, standing and sitting.

In 1994, a CP register and health care programme for children with CP, known as CPUP, was initiated in the southernmost counties of Swedish (Skåne and Blekinge) with a population of 1.3 million. A systematic search to find all children with CP and offer them the chance to participate in CPUP was performed in 1998, 2002 and 2006. The 1998 and 2002 prevalence of CP in children 4-7 years of age was 2.4/1000 and 2.6/1000, respectively [2-4].

The aim of this study was to analyse the development of lower limb passive ROM in relation to age, severity of gross motor function and CP subtype in children with CP.

## Methods

The inclusion criteria were children with CP born during 1990-1999 who were living in the area in the year during which they reached their third birthday and still living in the area in the year of their seventh birthday. Of the 393 children fulfilling these criteria, 359 (91%; 154 girls and 205 boys) participated in this study and the follow-up programme. In the majority of the 34 children fulfilling the study criteria but not participating in the CPUP programme, the CP diagnosis had recently been established. Young age and ataxic CP were more frequent in this group but with gender distribution identical to the study group. CP was defined according to the criteria described by Mutch *et al*. [5]. The CP subtype was determined after the fourth birthday according to the Surveillance of Cerebral Palsy in Europe (SCPE) network classification [6].

The gross motor function was classified according to the gross motor function classification system (GMFCS) [7], which is an age-related five-level system in which level I is the most and level V the least independent. The GMFCS level used in this study was the first level reported by the child's local physiotherapist after its fourth birthday.

The number of children in relation to CP subtypes and GMFCS level is presented in Table 1.

**Table 1:** Number of children in relation to cerebral pals (CP) subtypes gross motor function classification system (GMFCS) level.

**CP subtype**	**GMFCS I**	**GMFCS II**	**GMFCS III**	**GMFCS IV**	**GMFCS V**	**Total**
Spastic unilateral	103 (28.7%)	14 (3.9%)	5 (1.4%)	0 (0%)	0(0%)	122 (34.0%)

Spastic bilateral	55 (15.3%)	24 (6.7%)	32 (8.9%)	22 (6.1%)	22 (6.1%)	155 (43.2%)

Ataxic	10 (2.8%)	13 (3.6%)	8 (2.2%)	2 (0.6%)	1(0.3%)	34 (9.5%)

Dyskinetic	3 (0.8%)	1 (0.3%)	6 (1.7%)	14 (3.9%)	19 (5.3%)	43 (12.0%)

Non-classifiable	0 (0%)	0 (0%)	0 (0%)	2 (0.6%)	3 (0.8%)	5 (1.4%)

Total	171 (47.6%)	52 (14.5%)	51(14.2%)	40(11.1%)	45 (12.5%)	359 (100%)

In the follow-up programme, the child's local physiotherapist examined the child twice a year until 6 years of age, then once a year. The passive ROM was measured in stated and standardized ways. In the present study all assessments of hip abduction, hip external rotation, popliteal angle, knee extension and dorsiflexion of the foot with extended knee from the start 1994 until 1 January 2007 were included (Table 2). The measurements of hip extension were excluded due to a change of measurement methodology during the follow-up period.

**Table 2:** Goniometer positioning and standardization procedure for all five joint angles.

	**Extremity position**	**Goniometer:** **stationary arm**	**Goniometer:** **movable arm**	**End position**	**Additional standardization**
Hip abduction	Supine. Test leg in natural (extended position).	Along a line joining the two anterior superior iliac spines.	Parallel to longitudinal axis of femur.	Hip abducted to limit of motion	Pelvis stabilized by fixing opposite leg slightly abducted and flexed over edge of plinth.

Hip external rotation	Prone. With extended hips and the test leg knee flexed to 90°. Tester holding the tested leg and secure the pelvic rotation by stabilizing the pelvis with the other hand.	Parallel to the plinth.	Parallel to longitudinal axis of tibia.	External rotation to limit of motion just before pelvis just starts to lift from plinth.	

Popliteal angle	Supine. Test leg flexed to 90° hip and knee. Place one hand at the anterior aspect of the knee, and other at the distal calf, posteriorly.	Parallel to the sagittal plane of femur.	Parallel to the sagittal plane of tibia.	Knee extended to limit of motion. Estimate the degrees of the angle on the posterior side of the flexed knee. A fully extended knee is 180°.	Contralateral leg maintained in extension to stabilize the pelvis.

Knee extension	Supine with extended hips and knees.	Parallel to femur and trochanter major.	Parallel to tibia and the lateral malleol.	Knee extended to limit of motion. Extension deficit is reported with minus.	

Foot dorsiflexion	Supine. The knee extended.	Parallel to the longitudinal axis of fibula.	Parallel to the longitudinal axis of fifth metatarsal.	Foot dorsiflexed to limit of motion.	Hind foot maintained in neutral to avoid calcaneal valgus or varus.

The calculations are based on both lower extremities, except for the children with unilateral spastic CP. For the measurements related to CP subtypes, the five children with non-classifiable CP were excluded. The results are presented for the age period 2-14 years of age, as there are few measurements in the lowest and highest age groups. In total, the results are based on 5075 measurements (4939 measurements relating to the CP subtype). The number of measurements in relation to age, GMFCS levels and CP subtypes is presented in Tables 3 and 4.

**Table 3:** Number of children and measurements (in brackets) in relation to gross motor function classification system (GMFCS) level and age.

**Age**	**GMFCS I**	**GMFCS II**	**GMFCS III**	**GMFCS IV**	**GMFCS V**	**Total**
2	61 (128)	20 (47)	28 (81)	24 (76)	26 (76)	(408)

3	90 (194)	30 (70)	33 (100)	28 (84)	29 (82)	(530)

4	120 (271)	36 (99)	40 (124)	33 (108)	38 (122)	(724)

5	129 (276)	37 (100)	44 (129)	32 (106)	40 (118)	(729)

6	128 (205)	46 (85)	32 (67)	36 (80)	32 (76)	(513)

7	138 (214)	30 (55)	36 (81)	33 (68)	29 (66)	(484)

8	99 (155)	28 (56)	33 (67)	24 (52)	25 (54)	(384)

9	93 (146)	26 (47)	31 (61)	24 (56)	25 (58)	(368)

10	82 (131)	23 (40)	28 (53)	18 (38)	19 (42)	(304)

11	63 (99)	20 (34)	25 (53)	13 (28)	14 (34)	(248)

12	43 (64)	19 (40)	19 (35)	11 (26)	13 (34)	(199)

13	33 (48)	13 (24)	12 (21)	8 (16)	5 (10)	(119)

14	16 (22)	11 (19)	6 (12)	3 (6)	3 (6)	(65)

Total	(1953)	(716)	(884)	(744)	(778)	(5075)

Notice that each child can contribute more than one measurement at a specific age.

**Table 4:** Number of children and measurements (in brackets) in relation to cerebral palsy subtypes and age.

**Age**	**Ataxic**	**Dyskinetic**	**Spastic** **Unilateral**	**Spastic** **Bilateral**	**Total**
2	5 (12)	28 (84)	45 (70)	75 (230)	(396)

3	8 (24)	33 (98)	65 (96)	96 (292)	(510)

4	12 (32)	35 (118)	89 (142)	119 (406)	(698)

5	14 (40)	36 (98)	94 (143)	127 (414)	(695)

6	20 (42)	35 (80)	94 (111)	116 (262)	(495)

7	21 (44)	35 (72)	92 (100)	112 (256)	(472)

8	16 (34)	25 (50)	67 (70)	97 (222)	(376)

9	17 (34)	26 (60)	64 (68)	90 (202)	(364)

10	16 (32)	17 (34)	54 (58)	82 (178)	(302)

11	14 (28)	15 (36)	40 (42)	66 (142)	(248)

12	14 (28)	10 (20)	31 (33)	50 (118)	(199)

13	9 (18)	5 (10)	24 (25)	33 (66)	(119)

14	8 (16)	4 (8)	14 (15)	13 (26)	(65)

Total	(384)	(768)	(973)	(2814)	(4939)

Notice that each child can contribute more than one measurement at a specific age.

Of the 359 children, 59 had undergone a tendo Achilles lengthening (TAL) operation, 47 adductor-psoas tenotomy, 30 varus osteotomy of the proximal femur, six hamstring lengthening, 11 intrathecal baclofen pump (ITB) and 28 selective dorsal rhizotomy (SDR). Several operations had been performed in combination.

### Statistics

Initially, in order to assess the development of the population mean range of motion with age, non-parametric regression was used to approximate the functional form of the age-ROM relationship. The approximation showed that it is reasonable to view the development of mean ROM as being in a state of constant increase or decrease with a change in direction of this development at a specific age. The magnitude of the increase or decrease, as well as the specific age of change of development, was estimated using segmented regression. In the statistical software STATA 10 [8], this amounted to using non-linear regression together with STATA's indicator function. Because of the correlation structure imposed by the inclusion of both legs for most children, the estimated standard errors were calculated using STATA's robust estimates.

The analysis was first stratified on GMFCS level and then on CP subtype. The estimated ROM mean development with age was plotted in a graph together with corresponding point-wise confidence intervals for visual assessment. The point-wise confidence intervals were constructed as percentile intervals using a parametric bootstrap simulation. The simulation was based on the segmented regression estimates. In short, the process of constructing the point-wise intervals can be described as using the regression standard errors and means to produce different estimated means of ROM at a specific age. The different observed means then allow us to deduce information about the variance of the estimated mean and thereby construct the corresponding confidence intervals.

The study is based on an initial cross-sectional sample from a total population of children with CP measured repeatedly over time. Data holds information on both the individual development with age and the differences between the sub-groups included in the study at different ages. We also separated these effects by estimating them separately in accordance with the details given in a study by Fitzmaurice *et al*. [9]. Thereby, the estimate of the longitudinal age-effect is corrected for potential confounding with cross-sectional cohort effects, such as possible variation between treatments or other factors related to ROM development. This was done by allowing for different means of ROM for different birth cohorts, for example 1990-1991, 1992-1995 and 1996-1999. The predicted mean ROM for children born 1996-1999 is presented.

### Ethics

The study was approved by the Medical Research Ethics Committee at Lund University (LU-443-99). Informed consent from the parents of the children participating in the study was obtained.

## Results

In the total population of children with CP the mean range of hip abduction and external rotation, the popliteal angle the knee extension and the range of dorsiflexion of the foot decreased during 2-14 years of age (Figures 1, 2, 3, 4, 5). The results are adjusted for the possible effect of different birth cohorts. The ROM related to the GMFCS level and CP subtype is presented in Figures 6, 7, 8, 9, 10 and 11, 12, 13, 14, 15, respectively.

**Figure 1:**
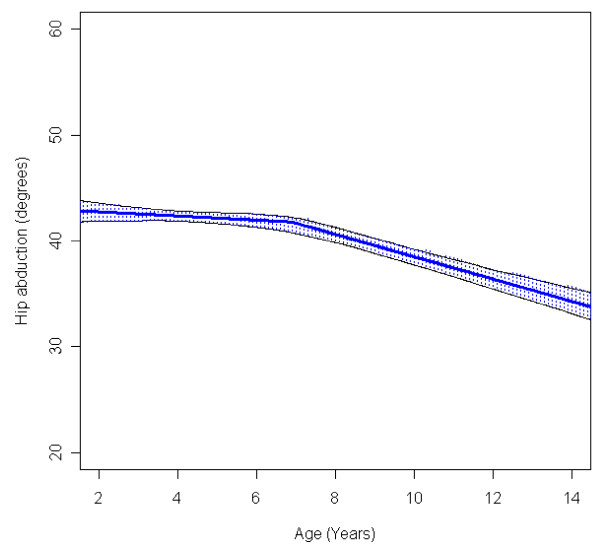
**Hip - abduction, mean range of motion (with 95% confidence interval) related to age at measurement in a total population of children with cerebral palsy**.

**Figure 2:**
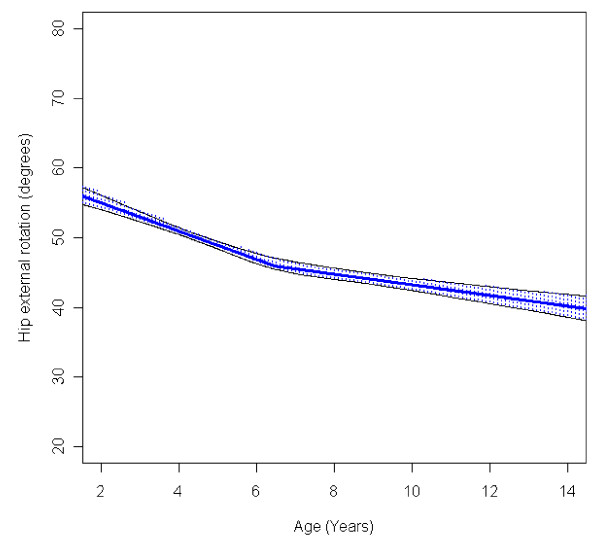
**Hip - external rotation, mean range of motion (with 95% confidence interval) related to age at measurement in a total population of children with cerebral palsy**.

**Figure 3:**
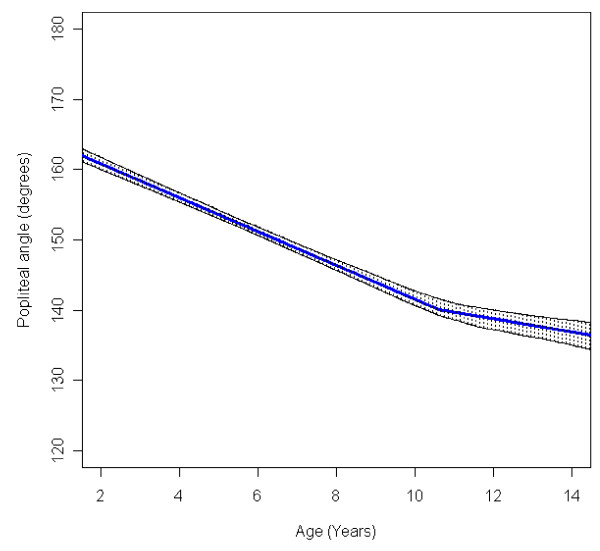
**Popliteal angle, mean range of motion (with 95% confidence interval) related to age at measurement in a total population of children with cerebral palsy**.

**Figure 4:**
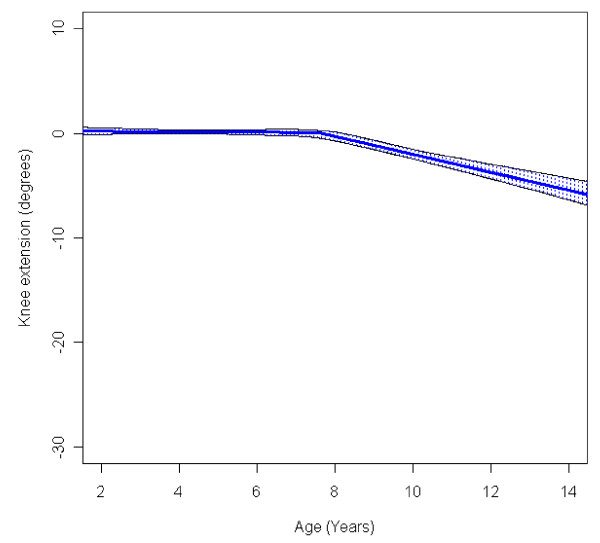
**Knee - extension, mean range of motion (with 95% confidence interval) related to age at measurement in a total population of children with cerebral palsy**.

**Figure 5:**
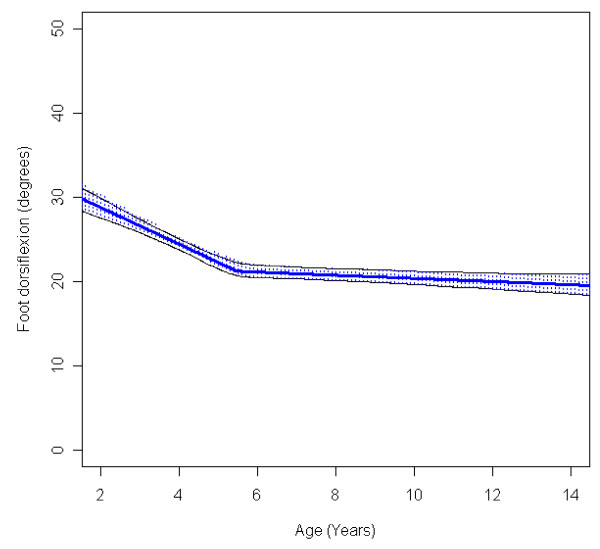
**Foot - dorsiflexion, mean range of motion (with 95% confidence interval) related to age at measurement in a total population of children with cerebral palsy**.

**Figure 6:**
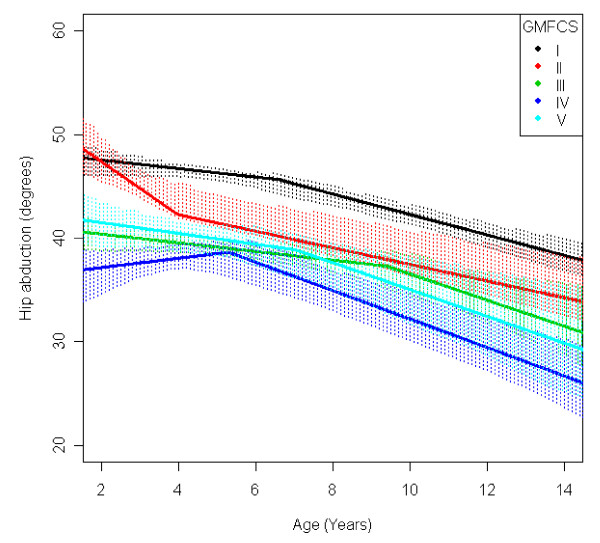
**Hip - abduction, mean range of motion (with 95% confidence interval) related to age at measurement and gross motor function classification system level in a total population of children with cerebral palsy**.

**Figure 7:**
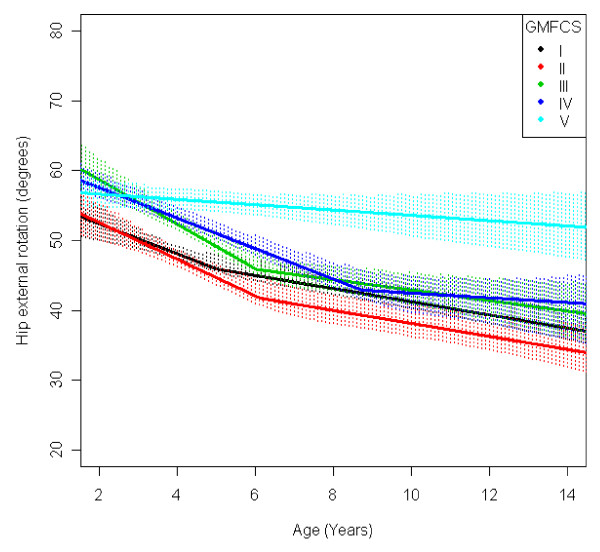
**Hip - external rotation, mean range of motion (with 95% confidence interval) related to age at measurement and gross motor function classification system level in a total population of children with cerebral palsy**.

**Figure 8:**
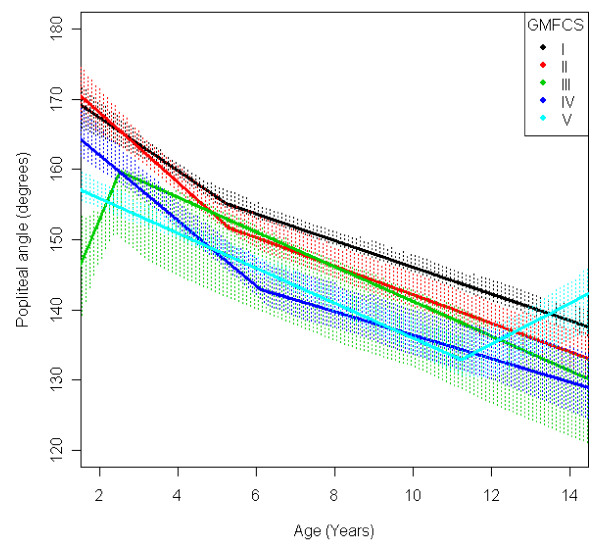
**Popliteal angle, mean range of motion (with 95% confidence interval) related to age at measurement and gross motor function classification system level in a total population of children with cerebral palsy**.

**Figure 9:**
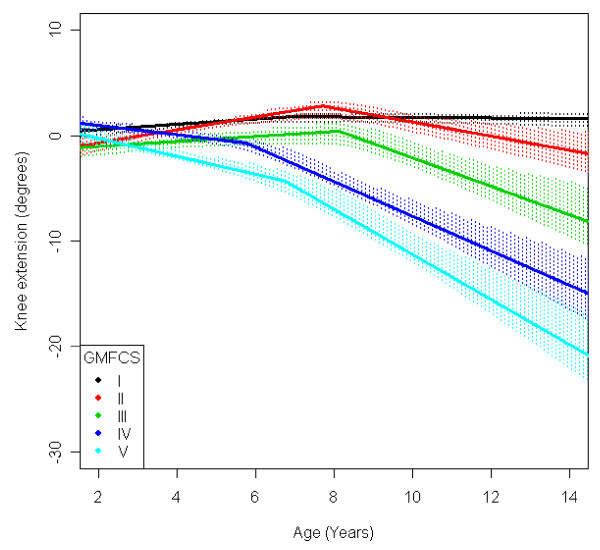
**Knee - extension, mean range of motion (with 95% confidence interval) related to age at measurement and gross motor function classification system level in a total population of children with cerebral palsy**.

**Figure 10:**
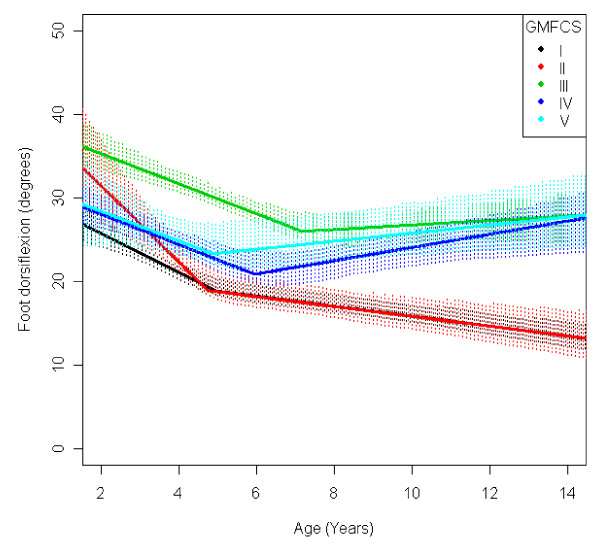
**Foot - dorsiflexion, mean range of motion (with 95% confidence interval) related to age at measurement and gross motor function classification system level in a total population of children with cerebral palsy**.

**Figure 11:**
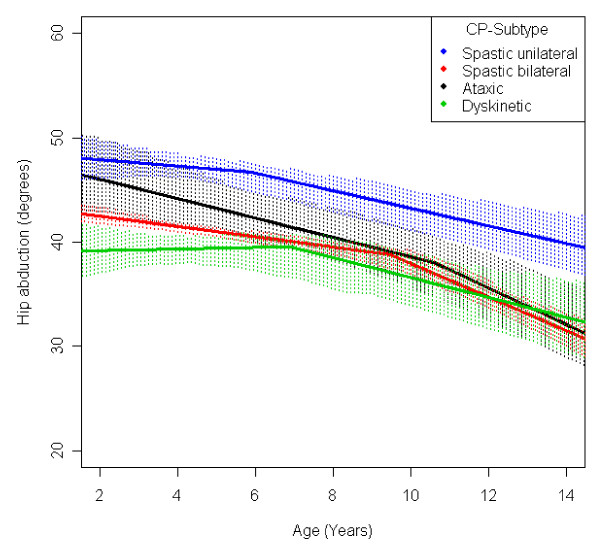
**Hip - abduction, mean range of motion (with 95% confidence interval) related to age at measurement and cerebral palsy (CP) subtype in a total population of children with CP**.

**Figure 12:**
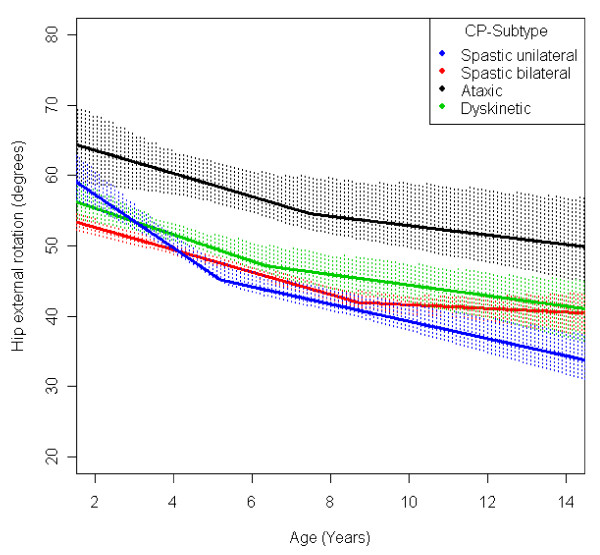
**Hip - external rotation, mean range of motion (with 95% confidence interval) related to age at measurement and cerebral palsy (CP) subtype in a total population of children with CP**.

**Figure 13:**
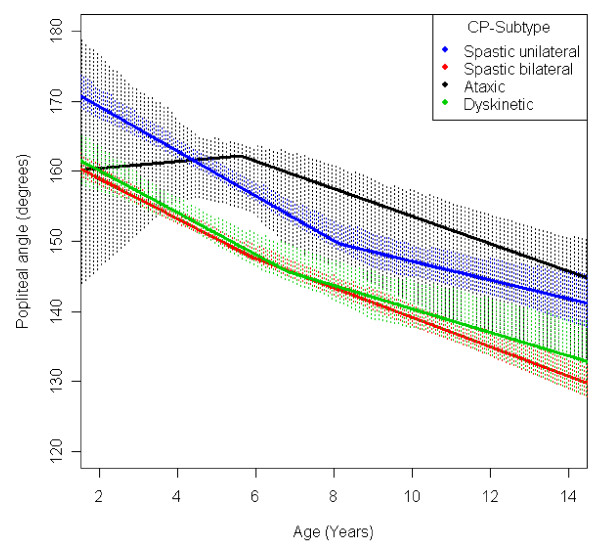
**Popliteal angle, mean range of motion (with 95% confidence interval) related to age at measurement and cerebral palsy (CP) subtype in a total population of children with CP**.

**Figure 14:**
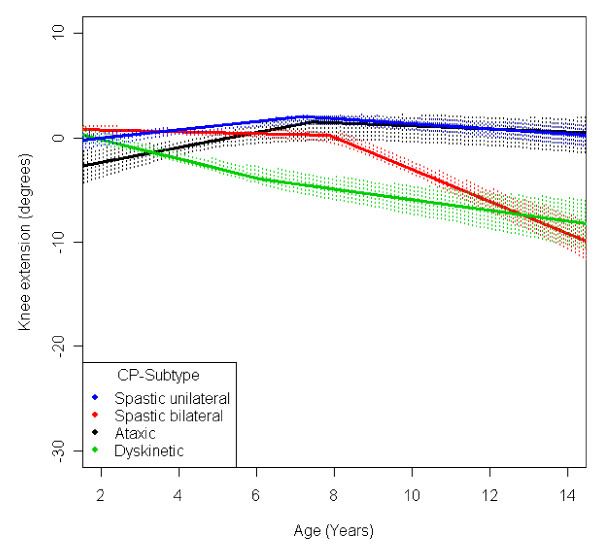
**Knee - extension, mean range of motion (with 95% confidence interval) related to age at measurement and cerebral palsy (CP) subtype in a total population of children with CP**.

**Figure 15:**
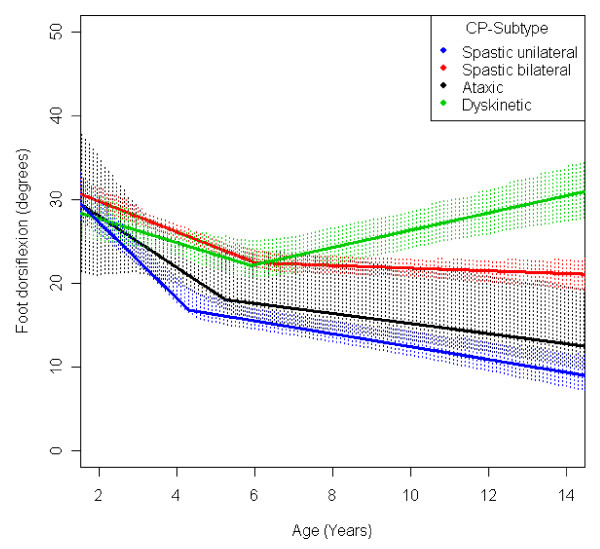
**Foot - dorsiflexion, mean range of motion (with 95% confidence interval) related to age at measurement and cerebral palsy (CP) subtype in a total population of children with CP**.

The mean range of hip abduction decreased from 43° to 34° (Figure 1). The decrease was more pronounced after 7 years of age. The range of abduction was higher in children with unilateral spastic CP (USCP) compared with the other CP subtypes (Figure 11), and in children in GMFCS I compared with children in GMFCS III-V (Figure 6). The exclusion of the 56 children who had undergone an adductor-psoas tenotomy or varus osteotomy of the femur did not change the results.

The mean range of external rotation of the hip decreased from 57° to 40° (Figure 2). The decrease was more pronounced before 7 years of age. Children in GMFCS V showed a higher degree of external rotation and no significant decrease with age (Figure 7). Children with ataxic CP had a higher degree of external rotation compared with other subtypes. Children with USCP had the lowest range of external rotation (Figure 12). Excluding the 56 children undergone adductor-psoas tenotomy or varus osteotomy of the femur did not change the results.

The mean popliteal angle decreased from 162° to 137° (Figure 3). The popliteal angle was higher in children with a higher level of gross motor function (Figure 8) and in children with ataxic CP and USCP compared with those with bilateral spastic CP (BSCP) and dyskinetic CP (Figure 13). The exclusion of the six children who undergone an operation for hamstring lengthening did not change the results. The increasing ROM for children in GMFCS V after 11 years of age is based on a few children (Table 3) and, therefore, is not likely to be representative.

The range of knee extension decreased by 6° during the age period studied (Figure 4). Children in GMFCS I group had a slight increase in knee extension during the period. Those in GMFCS II and III showed an increased ROM up to the age of 7-8 which was then followed by a decreasing range of extension. Children in GMFCS IV-V decreased during the whole period studied, and the decrease was more pronounced after 6-7 years of age (Figure 9). Differences in the mean knee extension between the GMFCS subgroups in the teenage period were statistically significant. Children with USCP and ataxic CP showed no significant change in knee extension; children with BSCP decreased after 6-7 years of age; and children with dyskinetic CP decreased over the entire study period (Figure 14). Our exclusion of the six children who had undergone a hamstring lengthening operation did not affect the results.

The mean range of dorsiflexion of the foot decreased from 30° to 20° up to 5 years of age and then remained almost equal during the remaining growth period (Figure 5). The decrease during the first years was seen in all levels of GMFCS and in all CP subtypes (Figures 10 and 15). Those at GMFCS levels I and II showed a further decrease with age, while those at GMFCS levels III-V increased their range of dorsiflexion after 5-7 years of age. Children with USCP and ataxic CP showed a further decrease in dorsiflexion after 5-7 years of age. Those with BSCP also decreased but at a higher level than those with USCP or ataxic CP. Children with dyskinetic CP improved their range of dorsiflexion after 6 years of age. The exclusion of the 59 children who had received tendo Achilles lengthening (TAL) treatment did not alter the results.

## Discussion

The present study is, to our knowledge, the first study of the development of lower limb passive ROM measured in a total population of children with CP. All children included were participating in CPUP, where the aim is to identify all children with CP or possible CP at an early stage. The diagnosis and the CP subtype are confirmed after the child's fourth birthday [4]. The proportion of children with the mildest gross motor functional limitation, GMFCS I, was higher than reported from most other studies, for example, from western Sweden and Victoria, Australia [10,11]. The reason for this might be that we have made active and systematic searches every fourth year to find children with undiagnosed CP, many of whom have less functional limitations than those who are more easily recognized. The GMFCS level used in this study was the first after 4 years of age, when the interrater reliability is better than in younger children [7].

In CPUP there is a heavy emphasis on practising and learning how to measure passive ROM with a goniometer in a standardized way. The importance of training and its impact on reliability has been demonstrated by Fosang *et al*. [12]. Measurement errors of 10°-15° have typically been reported for goniometric measures of one-joint muscles in children with CP [13-16]. However, in the present analysis, based on close to 5000 measurements, a low reliability, in the absence of systematic errors, would only increase the observed variation in the population ROM measurements, with increasing confidence interval width.

Since the development of ROM potentially differs between birth cohorts, the predicted mean ROM is presented only for children born during 1996-1999. This specific birth cohort was chosen in order to produce as current an estimate of development with age as possible. When comparing these results to those from other studies, one should bear in mind the distribution of GMFCS levels and CP subtypes in the study population, as well as their access to early and continuing services during their developing years.

The study showed a decreasing mean of ROM during the period of 2-14 years of age in all joints or muscles measured. The development of ROM varied according to GMFCS level and CP subtype.

The decrease in external rotation of the hip, popliteal angle and dorsiflexion of the foot were more pronounced during the first 5-10 years of age (Figures 2, 3, 5). The decrease in hip abduction and knee extension were more pronounced after 7 years of age (Figures 1, 4).

Children with unilateral spastic CP showed a lower range of outward rotation of the hip and dorsiflexion of the foot than the other subtypes. However, they showed no decrease in knee extension and they had a higher than average range of hip abduction (Figures 11, 12, 14, 15). This corresponds well to the typical gait in children diagnosed with unilateral CP who have equinus foot, stiff knee and internal rotation of the hip [17].

Children with bilateral spastic and dyskinetic CP showed the lowest range of popliteal angle and knee extension, but they had the highest range of dorsiflexion of the foot (Figures 13, 14, 15). This corresponds with the known tendency of crouch gait in children with bilateral CP [18].

Children with ataxic CP showed the highest range of external hip rotation, popliteal angel and knee extension (Figures 12, 13, 14). They also had a lower than average dorsiflexion of the foot (Figure 15). This matches the gait pattern of children with ataxic diplegia (almost half of the children with ataxic CP) who have hypotonia and some distal spasticity and often stand and walk with hyperextended knees (genu recurvatum).

The development of ROM related to the GMFCS level showed a more pronounced decrease in hip abduction, popliteal angle and knee extension in children with lower levels of gross motor function (Figures 6, 8, 9). This is seen mainly in the development of children with bilateral spastic and dyskinetic CP. The decrease in outward hip rotation and dorsiflexion of the foot was more pronounced in children with higher levels of gross motor function (Figures 7, 10), which is seen mainly in children with unilateral spastic CP and ataxia.

Growth of the length of a muscle is stimulated by the growth of the length of the skeleton and by the muscle excursion [19]. Spasticity may result in reduced muscle excursion, leading to failure of muscle growth, with contracture seen as restricted ROM [20]. Reduced muscle excursion due to muscle weakness, inability to stand or walk also contributes to contracture development.

The speed of growth is highest during the first years of age, which could explain the more rapid decrease in ROM during these years. A recent study (based mainly on the same material as the present study) showed an increasing tone of the gastrocnemius muscle in children with CP up to 4 years of age, followed by a decreasing muscle tone up to 12 years of age [21]. These findings could be one explanation for the decreased progress of contracture development after 4-5 years of age.

A decrease in ROM with age may result in decreased mobility and a further decrease in muscle excursion - a vicious circle. Decreased mobility may lead to activity limitation and participation restrictions [22-24]. This is one reason for the continuous standardized follow-up of ROM in CPUP, as it enables early identification and treatment of decreasing ROM interfering with function. Although this study showed a decrease in ROM with age, earlier studies have shown that CPUP has decreased the development of severe contractures in children with CP and reduced the need for operative treatment of contractures [25,26].

The present study does not show the natural course of ROM, as the children have been given treatment to prevent the development of severe contractures. However, excluding the children who had undergone orthopaedic operations did not significantly change the results. As only a few children had undergone tonus-reducing operations, SDR or ITB, these treatments should not have influenced the results.

## Conclusion

We found a decrease in ROM from 2-14 years of age in children with CP. The development of ROM varied with age and according to GMFCS level and CP subtype. Having knowledge of the development in a total population is of value to planning health care programmes for children with CP and in the analysis of future prognostic developments of ROM related to an individual's CP subtype and GMFCS level. It is also a useful reference for future intervention studies.

## Abbreviations

BSCP: bilateral spastic CP; CP: cerebral palsy; CPUP: Swedish health care programme for children with cerebral palsy; GMFCS: gross motor function classification system; ITB: intrathecal baclofen pump; ROM: range of motion; SCPE: Surveillance of Cerebral Palsy in Europe; SDR: selective dorsal rhizotomy; TAL: tendo Achilles lengthening; USCP: unilateral spastic cp (spastic hemiplegia).

## Competing interests

The authors declare that they have no competing interests.

## Authors' contributions

All the authors designed the study. PW performed the statistical analysis. All authors analysed the results, contributed to the draft manuscript, read and approved the final manuscript.

## Pre-publication history

The pre-publication history for this paper can be accessed here:


