# Development of lower limb range of motion from early childhood to adolescence in cerebral palsy: a population-based study

**DOI:** 10.1186/1741-7015-8-49

**Published:** 2010-07-28

**Authors:** Eva Nordmark, Gunnar Hägglund, Henrik Lauge-Pedersen, Philippe Wagner, Lena Westbom

**Affiliations:** 1 Department of Health Sciences, Division of Physiotherapy, Lund University, SE-221 00 Lund, Sweden; 2 Hospital for Children and Adolescents, Lund University Hospital, SE-221 85 Lund Sweden; 3 Department of Orthopaedics, Lund University Hospital SE-221 85 Lund, Sweden; 4 National Competence Centre for Musculoskeletal Disorders, Lund University Hospital, SE-221 85 Lund, Sweden; 5 Department of Clinical Sciences, Division of Paediatrics, Lund University, Lund, Sweden

## Correction

After the publication of this work [1], we became aware of the fact that the description of the knee position for one of the measurements (dorsiflexion of the foot) was not correctly described. It was described as dorsiflexion of the foot with extended knee. The correct description is dorsiflexion of the foot with knee flexion. The presented results, discussion and conclusion are based on the position used (knee in flexion).

In the method section, see following paragraph: "In the present study all assessments of hip abduction, hip external rotation, popliteal angle, knee extension and dorsiflexion of the foot with knee flexion from the start 1994 until 1 January 2007 were included (Table 2)."

In Table 2. Goniometer positioning and standardization procedure for all five joint angles, Extremity position Foot dorsiflexion. Supine. Knee in flexion.

## Pre-publication history

The pre-publication history for this paper can be accessed here:

http://www.biomedcentral.com/1741-7015/8/49/prepub
